# TEAD4 exerts pro‐metastatic effects and is negatively regulated by miR6839‐3p in lung adenocarcinoma progression

**DOI:** 10.1111/jcmm.13634

**Published:** 2018-04-18

**Authors:** Qun Zhang, Hang Fan, Qian Zou, Hongda Liu, Bing Wan, Suhua Zhu, Yangbo Hu, Huijuan Li, ChenXi Zhang, Li Zhou, Qingqing Zhu, Kunhong Xiao, Jianya Zhang, Ping Zhan, Tangfeng Lv, Yong Song

**Affiliations:** ^1^ Department of Respiratory Medicine Jinling Hospital Nanjing University School of Medicine Nanjing China; ^2^ Department of Pharmacology and Chemical Biology School of Medicine University of Pittsburgh Pittsburgh PA USA; ^3^ Department of ICU the Affiliated Hospital of Jiangsu university Zhenjiang China; ^4^ Department of Respiratory Medicine Jinling Hospital Southeast University School of Medicine Nanjing China; ^5^ Department of Respiratory Medicine Jinling Hospital Jinling Clinical Medical College of Nanjing Medical University Nanjing China; ^6^ Department of Respiratory Medicine and Central Laboratory Nanjing Chest Hospital School of Medicine Southeast University Nanjing China; ^7^ Department of Respiratory Medicine The First Affiliated Hospital of Soochow University Suzhou China

**Keywords:** invasion, lung adenocarcinoma, miR6839‐3p, prognosis, TEAD4

## Abstract

Several studies have shown the tumorigenesis role of transcriptional enhancer associate domain (TEAD) proteins; here, we initially explored expression, function and signalling mechanisms of TEAD4 in lung adenocarcinoma (LAD). Both the mRNA and protein levels of TEAD4 were increased in LAD tissues than those in adjacent nontumourous tissues. Besides, database search indicated a poorer clinical outcome in LAD patients with higher TEAD4 expression, revealing its potential tumour‐promoting role. Therefore, we conducted cellular experiments to further investigate its effect on tumour phenotypes. Accordingly, TEAD4 showed little impact on LAD cell cycle, proliferation, or apoptosis. However, silencing TEAD4 remarkably attenuated cell migration and invasion capacities. Consistently, several important epithelial‐mesenchymal transition (EMT) markers such as E‐cadherin and Slug were consequently altered by silencing TEAD4. Furthermore, we identified a novel TEAD4‐targeted microRNA, namely miR6839‐3p, and confirmed its function in suppressing TEAD4 expression. Finally, the impact of overexpressing miR6839‐3p mimics on LAD progression was validated, which showed a similar pattern with TEAD4 knockdown cells. Taken together, our data not only revealed the significant role of TEAD4 in promoting LAD progression and predicting clinical outcome but also distinguished miR6839‐3p mimics as a promising therapeutic direction.

## 1 INTRODUCTION

Lung cancer is one of the most prevalent malignant tumours with the highest growth rate of morbidity and mortality, currently is the leading cause of cancer‐associated death and the greatest threat to human health.^1^ Histological classification of lung cancer includes 2 major subtypes: non‐small‐cell lung cancer (NSCLC) and small‐cell lung cancer (SCLC). The former one comprises approximately 80% of all lung cancer cases,^2^ including lung adenocarcinoma (LAD, ˜48% of NSCLC), lung squamous cell carcinoma (LSCC, ˜28%), and large cell carcinoma (LCC, ˜24%).^3^ LAD is the most common pathological type, with high risk of distant metastasis. Importantly, the high mortality rate of lung cancer is predominantly due to direct or indirect consequence of tumour dissemination to other organs.^4^ Despite significant advances in early detection and targeted therapies for lung cancer have been achieved, patients with advanced tumour stages (with distant metastasis) still end with poor clinical outcome. A further understanding of the cellular mechanisms that regulate NSCLC invasion and metastasis is therefore essential for developing novel and more effective therapeutic interventions.

The evolutionarily conserved Hippo protein is initially identified as a key regulator in various biological processes including cell proliferation, cell contact inhibition, and cancer development.^5^ Human ortholog of Hippo includes MST1 and MST2, and the corresponding signalling pathway generally functions in a tumour‐suppressing pattern by a kinase cascade.^6^ Briefly, MST1/2 phosphorylate and activate LATS1 (large tumour suppressor 1) and LATS2. Activated LATS1/2 then phosphorylate the YAP (YES‐associated protein 1) and TAZ transcriptional coactivators. Phosphorylation of YAP/TAZ will be degraded in cytoplasm instead of functioning in nucleus, therefore suppressing transcription events.^7^ The most important transcription factors downstream of YAP/TAZ are the TEAD (transcriptional enhancer associate domain) proteins. Four highly conserved TEAD members (TEAD1, TEAD2, TEAD3 and TEAD4) have been identified in mammals, all containing a YAP/TAZ interaction domain and a TEA/ATTS DNA‐binding domain.^8^ Besides YAP/TAZ, vestigial‐like (VGLL) proteins and p160 can also interact with the YAP/TAZ interaction domain on TEADs.^9^, ^10^ Of note, TEADs have been reported to be involved in tumour progression, including hepatocellular carcinoma,^11^ cholangiocarcinoma,^12^ Ewing sarcoma,^13^ gastric adenocarcinoma,^8^ and ovarian cancer.^14^ Interestingly, although the detailed roles of TEADs in lung cancer have not been clearly described, its coactivators were demonstrated in regulating lung malignancy. For example, the nucleus accumulation of YAP is associated with poor NSCLC overall survival.^15^ In contrast, VGLL4 competes with YAP in binding to TEADs and suppresses lung cancer progression.^3^ The tumour regulating role of TEAD coactivators triggered us to explore expression and function of TEADs in lung cancer.

Here, we firstly revealed that the expression level of TEAD4 in LAD was higher than that in normal lung tissues. Statistical analyses confirmed its predictive role for unfavourable clinical outcome of patients with LAD. Although cellular studies showed no significant effect of TEAD4 on LAD proliferation or apoptosis, silencing TEAD4 substantially attenuated the migration and invasion capacities of LAD cells by promoting EMT (epithelial‐mesenchymal transition) process. Of note, we initially identified a novel microRNA targeting TEAD4, miR6839‐3p, in LAD cells. The inhibitory effects of on TEAD4 signalling were also confirmed in our study, which may help direct the targeted therapeutic strategy for LAD patients with high TEAD4 expression.

## 2 METHODS

### 2.1 Patients and tissue samples

A total of 21 NSCLC tissue samples and paired adjacent normal lung tissue were obtained from Jinling Hospital (Nanjing, Jiangsu, China) between March 2015 and March 2016. Each patient signed an informed consent, and this study was approved by the Ethic Committee and Institutional Review Board of Jinling Hospital.

### 2.2 Tissue microarray (TMA) and immunohistochemical (IHC) staining

A TMA containing 87 LAD tissue samples and matched adjacent normal lung tissues (HLug‐Ade180Sur‐02; Shanghai Outdo Biotech, Shanghai, China) was used to evaluate the expression profile of TEAD4 by IHC. Briefly, TMA slides were sequentially subjected to deparaffinization, rehydration, blockage, antigen retrieval, primary antibody incubation (mouse monoclonal Anti‐TEAD4 antibody, SAB1404467; Sigma‐Aldrich, St. Louis, MO, USA), DAB staining and haematoxylin counterstaining as described before.^16^ The TEAD4 staining results were assessed and scored by 2 independent pathologists, based on both staining intensity and percentage of positive staining cells.^17^ Particularly, TEAD4 is located in both cytoplasm and nucleus; therefore, we separately evaluated the immunoreactivity in cytoplasm and nucleus (score 0‐12, respectively). The final IHC score (range 0‐24) was obtained by adding cytoplasm staining score and nucleus staining score.

### 2.3 Cell culture

Six human‐originated LAD cell lines (A549, SPC‐A1, NCI‐H1299, H1650, PC9 and H1975), one LSCC cell line (NCI‐H1703) and the human normal bronchial epithelial cell line (HBE) were purchased from The Institute of Biochemistry and Cell Biology of the Chinese Academy of Sciences (Shanghai, China). A549, SPC‐A1, NCI‐H1299, H1650 and NCI‐H1703 cells were maintained in RPMI 1640 medium supplemented with 10% foetal bovine serum (FBS, GIBCO, Gaithersburg, MD) and 1% penicillin‐streptomycin. HBE and PC9 cells were cultured in DMEM medium containing 10% FBS and 1% penicillin‐streptomycin. All cells were maintained at 37°C in a humidified atmosphere with 5% CO_2_.

### 2.4 MicroRNA (miRNA) and small interfering RNA (siRNA) transfection

The miRNA mimics, including has‐miR‐6839‐3p, has‐miR‐6780a‐3p, has‐miR‐375, has‐miR‐4269, has‐miR‐1343‐3p and negative control, were purchased from RiboBio (Guangzhou, China). Lipofectamine 2000 (Invitrogen, Shanghai, China) was employed to silence transcripts using TEAD4 siRNA (Genepharma, Shanghai, China) at a 60%‐80% cellular confluence. RiboFECTTM CP (RiboBio) was used to facilitate miRNA transfection according to the manufacturer's instructions. At 24‐48 hours post‐transfection, cells were subjected to quantitative PCR, Western blot and functional assays.

### 2.5 Cell proliferation and colony formation assays

MTT (3‐[4, 5‐dimethylthiazol‐2‐yl]‐2, 5‐diphenyl‐tetrazolium) assay was carried out in 96‐well plates at a density of 3000 cells/well after transfection. Cell proliferation was monitored every 24 hours with 20 μL MTT solution (5 mg/mL) added to each well and incubated for 4 hours and then adding 150 μL dimethyl sulfoxide to fully solubilize the MTT after removing the medium. The absorbance value was measured at 570 nm wavelength.^18^

Colony formation assay was performed on a 6‐well plate for 2 weeks. The colonies were fixed with paraformaldehyde (PFA) and followed by staining with 0.1% crystal violet (Sigma‐Aldrich). The visible colonies were manually counted. All experiments were performed at least 3 times.

### 2.6 Cell migration and invasion assays

Transwell assays were performed using polycarbonate transwell chambers (Corning Costar Corp., Cambridge, MA, USA). For invasion assay, the chambers were pre‐coated with 50 μL of 2.5 mg/mL Matrigel (BD Biosciences, Franklin Lakes, NJ, USA) and left to polymerize for 4‐6 hours at 37°C. Transfected cells were then seeded into the upper chambers with culture medium containing 1% FBS and allowed to migrate or invade for 24 hours at 37°C. The lower chambers were stocked with culture medium containing 20% FBS. The cells on the membrane were fixed with paraformaldehyde and stained with 0.1% crystal violet (Sigma). Cells were counted by a microscope from 5 casually random fields.^19^ Each experiment was performed in triplicate.

### 2.7 Flow cytometric analyses for apoptosis and cell cycle

The cultured cells were double stained with annexin‐V‐FITC and propidium iodide (PI) according to the manufacturer's instructions, and the flow cytometric analysis was performed by a BD FACScan® flow cytometry (BD Biosciences) followed by quadrant statistical analysis for the detection of early and late apoptotic cells, dead cells and viable cells. The ratio of early apoptotic cells and late apoptotic cells was compared to that of controls in each experiment.

For the cell cycle analysis, after fixing cells with 70% cold ethanol (700 μL) at 4°C overnight, the cells were washed and stained with PI using the CycleTESTTM PLUS DNA reagent kit (BD Biosciences) according to the manufacturer's protocol. The ratio of cells in various phases (G0, G1, S and G2/M phases) of cell cycles was counted and analysed. All experiments were performed at least 3 times.

### 2.8 Quantitative reverse transcription‐PCR

Total RNA was extracted from NSCLC or adjacent tissues or cultured cells using TRIZOL reagent (Invitrogen, Carlsbad, CA, USA) according to the manufacturer's instructions. A total amount of 1 μg RNA was reversely transcribed into cDNA using a commercial RT‐PCR kit (RR036A; TaKaRa, Dalian, China) according to the manufacturer's instructions. Total miRNA was synthesized using miRNA first‐strand cDNA Synthesis (tailing reaction) applied by Sangon Biotech (Shanghai, China) following manufacturer's protocols. Expression levels were quantified using a ABI 7500 real‐time PCR System (Applied Biosystems, Foster City, CA, USA) and SYBR Premix Ex Taq II (perfect real‐time) (RR420A; TaKaRa). The PCR reaction was conducted at 95°C for 30 seconds, followed by 40 cycles of 95°C for 5 seconds, and 60°C for 35 seconds. The primers used are presented in Table S1. All measurements were assessed with the 2^−∆∆CT^ method normalized by β‐actin for mRNA and U6 for miRNA.^20^

### 2.9 Western blotting

Harvested cells were homogenized in RIPA buffer supplemented with protease inhibitor cocktail at 4°C for 30 minutes. After centrifuged at 13000 g for 15 minutes, the protein concentration was tested by a Pierce BCA kit (Thermo Fisher Scientific). Approximately 30 μg proteins were isolated by SDS‐PAGE, transferred to a PVDF membrane, probed with primary antibodies followed by incubating with HRP‐conjugated secondary antibodies as described before.^21^ Enhanced chemiluminescence (ECL) was finally added to detect specific band. The immunoreactivity was semi‐quantified using Image J Software and GraphPad Prism 6.0 Software. Information on the antibodies used is presented in the Table S2.

### 2.10 Luciferase activity assays

H1299 cells were seeded in 24‐well plates at a density of 10^4^ cells/well. Upon reaching 70% confluence, each well was transiently cotransfected with either 1 μg GV272‐TEDA4‐WT (wild‐type) or 1 μg GV272‐TEDA4‐MUT (mutant) plasmids containing firefly luciferase, together with miR‐6839‐3p mimics or negative control as well as 10 ng endogenous control vector pRL‐SV40. Lipofectamine 2000^®^ was used for H1299 cells transfection with plasmids and mimics. The value of relative luciferase activity was measured by the dual luciferase assay system (Promega Corporation, Madison, WI, USA) according to the manufacturer's instructions.

### 2.11 Statistical analysis

Quantitative data were presented as the mean ± SD of at least 3 times in triplicate. All statistical evaluations were performed using the IBM SPSS Software (version 22.0; Chicago, IL, USA). The IHC results were plotted by Chi‐squared test and Spearman's rank correlation test. Univariate survival analyses were performed by Kaplan‐Meier procedure and subsequently estimated by the log‐rank test. Statistical differences were compared by Student's *t* test between the experimental and control groups of cellular results. *P *<* *.05 was considered to be statistically significant in all analyses.

## 3 RESULTS

### 3.1 TEAD4 is up‐regulated in NSCLC

**Figure 1:**
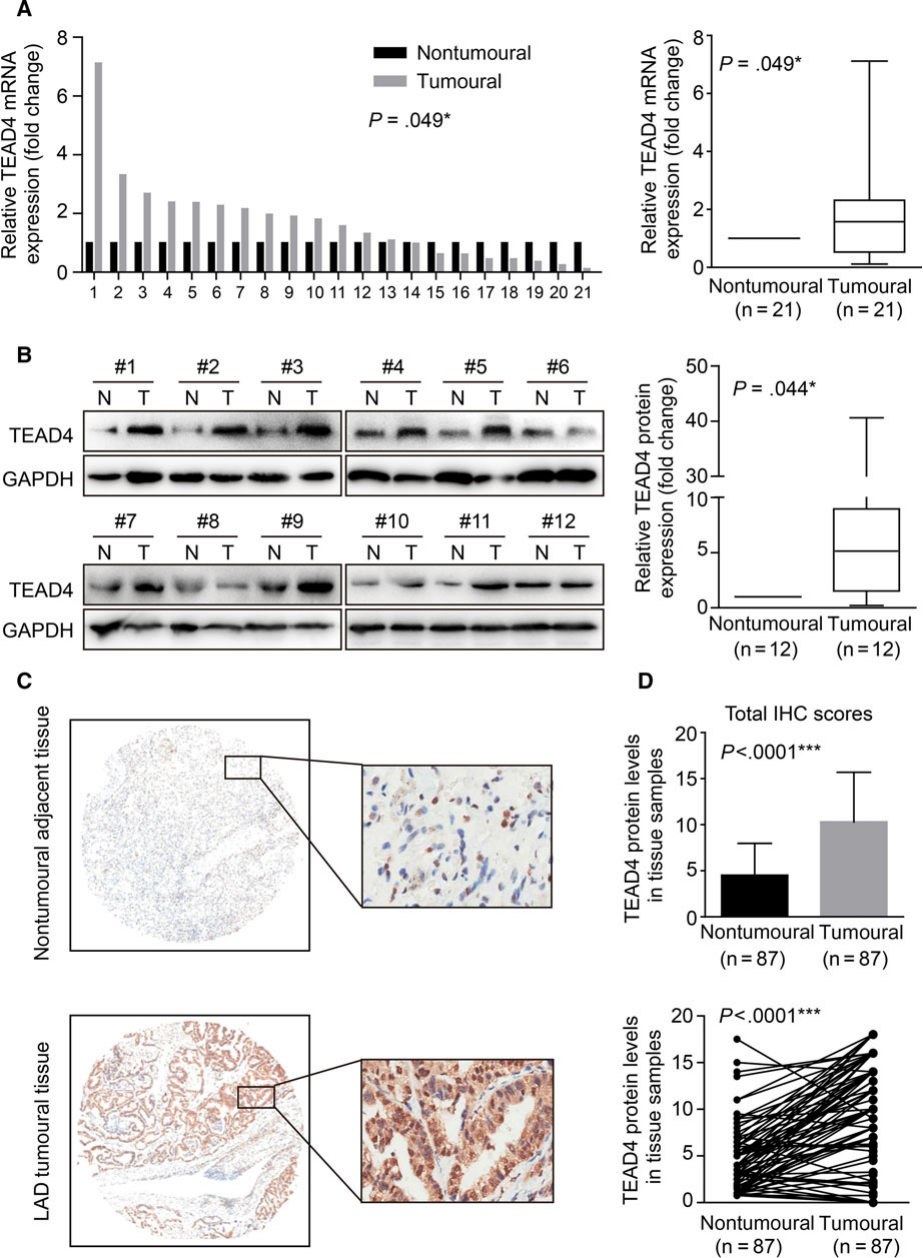
TEAD4 is up‐regulated in lung adenocarcinoma tissues. A, Real‐time PCR quantification of TEAD4 mRNA levels in lung adenocarcinoma (LAD) tissues and matched adjacent nontumoural tissues (n = 21). The mRNA expression of TEAD4 in LAD tissues was significantly higher than those in nontumoural tissues. B, Western blotting analysis of TEAD4 in 12 paired LAD tissues. Quantitative of relative protein levels were presented in the right panel. C, Representative immunohistochemical (IHC) results of TEAD4 protein expression in paired LAD tissues and adjacent nontumoural tissues, showing a positive staining in both nucleus and cytoplasm. D, Statistical analysis of TEAD4 immunoreactivity in 87 pairs of LAD tissues and adjacent nontumoural tissues. *P <* *.05, ***P <* *.001 compared to nontumoural group

We firstly tested the transcription level of TEAD4 by qRT‐PCR in a total of 21 pairs of clinical NSCLC tissues and adjacent healthy tissues. TEAD4‐mRNA levels were significantly higher in the cancerous tissues (Figure 1A, *P *=* *.049) from 13 patients (13/21, 61.9%). Besides, Western blot assay showed an up‐regulated level of TEAD4 protein in 12 (12/21, 57.1%) NSCLC tissues compared with that in adjacent normal tissues (Figure 1B, *P *=* *.044).

We then performed the IHC experiments on a tissue microarray containing 87 paired lung tissues and adjacent tissues. Immunostaining showed a predominantly cytoplasm location of TEAD4 along with slight nucleus staining (Figure 1C). Based on the immunoreactivity in both cytoplasm and nucleus, we evaluated the total TEAD4 protein expression scores (range 0‐24). Accordingly, the protein expression level of TEAD4 in LAD was significantly higher than that in the paired nontumourous tissues (Figure 1D, *P *<* *.0001), indicating the possible role of TEAD4 as an oncogenicity protein.

### 3.2 High TEDA4 expression is an unfavourable prognostic factor for NSCLC

**Figure 2:**
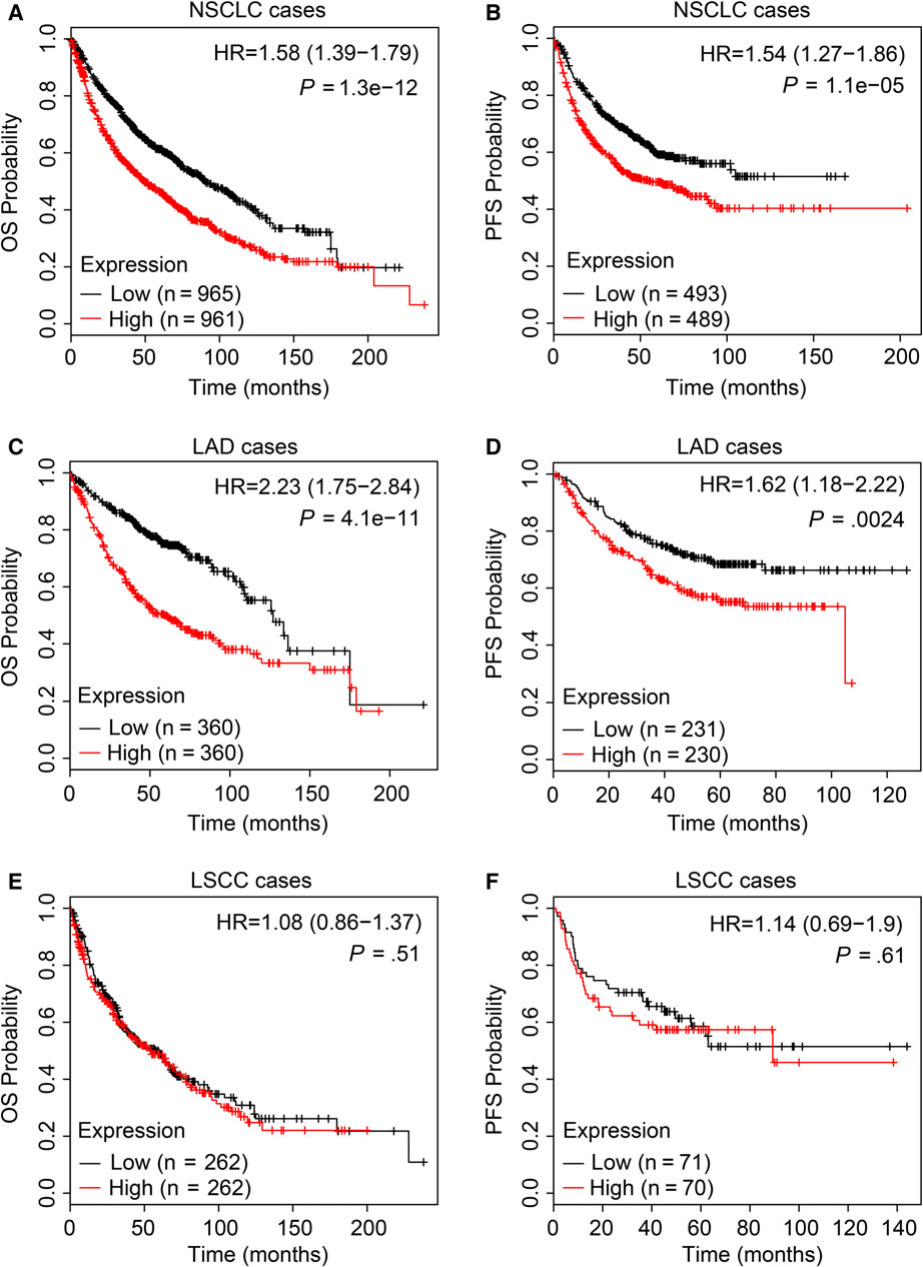
Kaplan‐Meier survival analyses of the overall survival (OS) and progression‐free survival (PFS) of patients with lung cancer. High TEAD4 accumulation was significantly correlated with poor OS (A) and PFS (B) of patients with NSCLC according to the Kaplan‐Meier analysis. Specifically, TEAD4 may help predict the clinical outcome of patients with LAD (C, D), although showed no statistical significance in its correlation with LSCC histological type (E, F)

To better investigate the clinical significance of TEAD4 in NSCLC, we evaluated its prognostic effect via a public database of Kaplan‐Meier plotter analysis (http://www.kmplot.com). The data demonstrated that a higher TEAD4 level was correlated with poorer overall survival (OS) and progression‐free survival (PFS) in NSCLC (Figure 2A,B; both *P *<* *.001). We then focused more on LAD cases, which showed a remarkable role of higher TEAD4 in predicting poorer clinical outcome (Figure 2C,D; both *P *<* *.05). In contrast, there seemed no predictive significance of TEAD4 in patients with LSCC from either OS or PFS (Figure 2E,F; both *P *>* *.05). Together, the data showed a promising role of TEAD4 in help predicting clinical outcome of patients with LAD.

### 3.3 Silencing TEAD4 shows little effect on tumour cell proliferation, cell cycle or apoptosis

**Figure 3:**
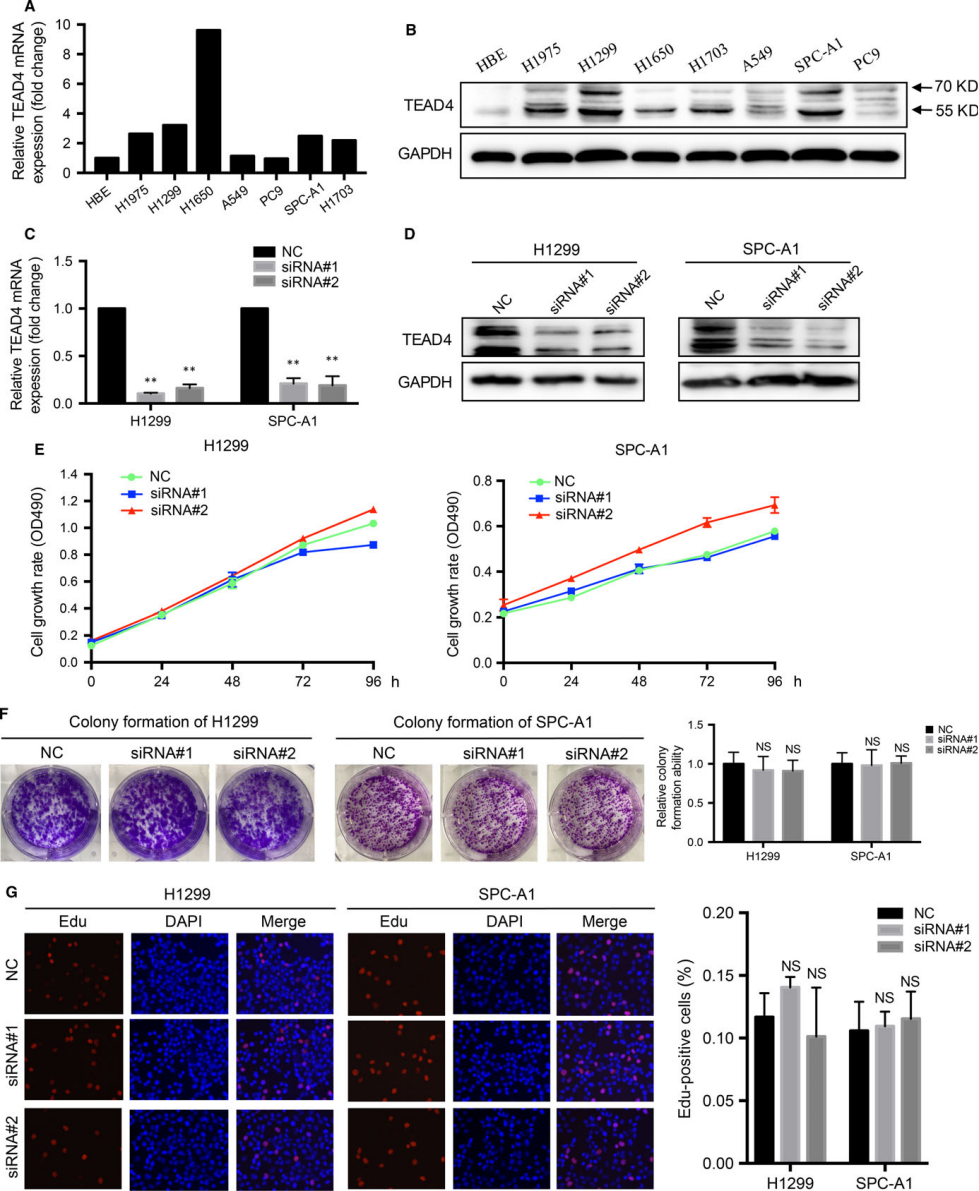
Silencing TEAD4 exerts no effect on proliferation of LAD cells. Quantitative PCR (A) and Western blot (B) were used to measure the TEAD4 levels in human bronchial epithelial cell line (HBE) and NSCLC cell lines (A549, H1975, H1299, H1650, A549, PC9, SPC‐A1 and H1703), indicating higher TEAD4 levels in NSCLC cells. The H1299 and SPC‐A1 cells were transfected with TEAD4‐siRNA, and transfection efficiencies were verified by qRT‐PCR (C) and Western blot (D), respectively. Silencing TEAD4 showed no significant effect on cellular proliferation as revealed by MTT (E), colony formation (F) and Edu staining (G) assays. The data were represented as the mean ± SD acquired from 3 repeated experiments. **P <* *.01 compared to negative control (NC) group

**Figure 4:**
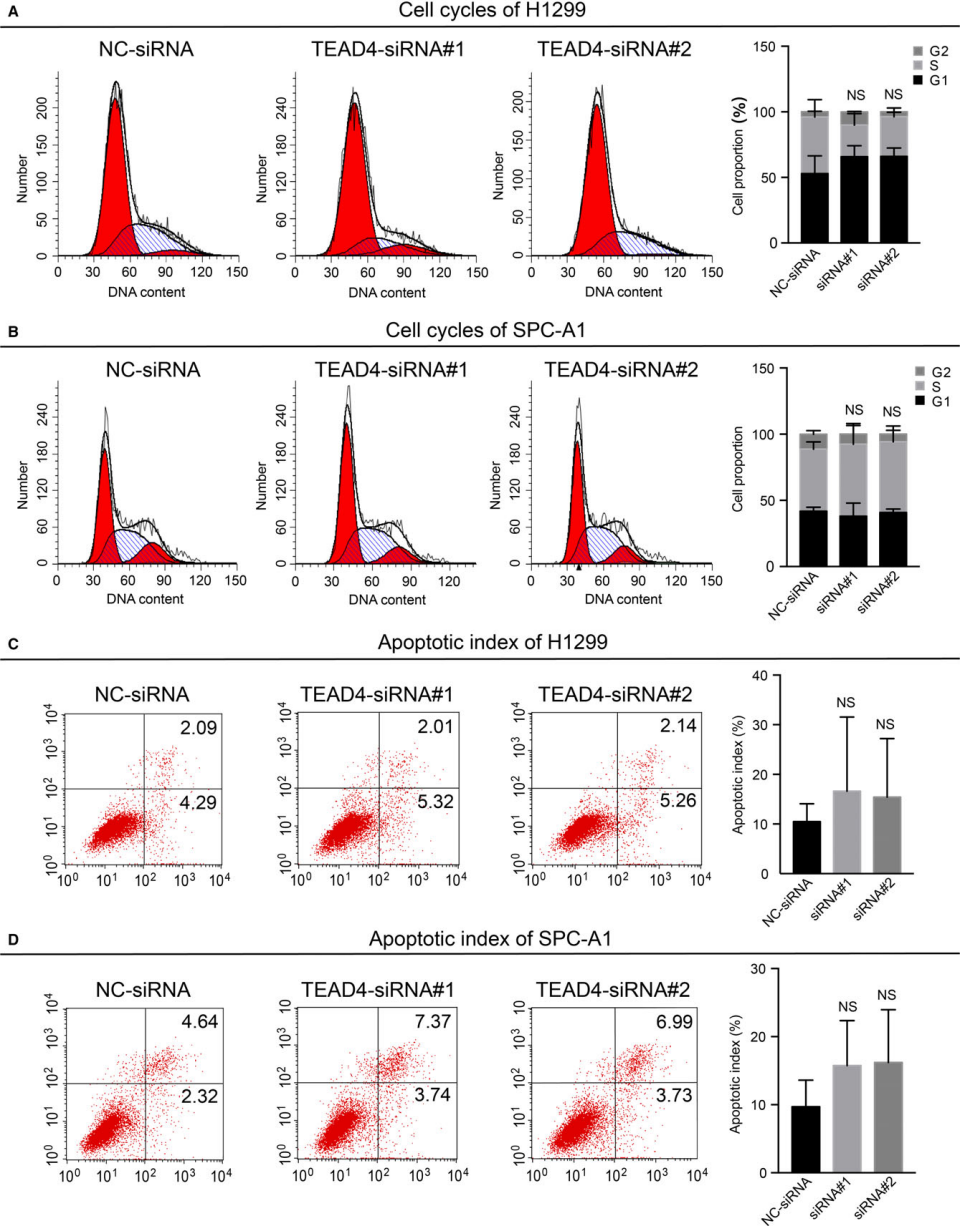
Down‐regulation of TEAD4 shows no influence on cell cycle or apoptosis of LAD cells. Cell cycle analyses were determined via flow cytometry assay for H1299 (A) and SPC‐A1 (B) cells transfected with TEAD4‐siRNA, and cells transfected with scramble siRNA were used as a negative control (NC). Cell apoptosis was also evaluated by flow cytometry strategy in H1299 (C) and SPC‐A1 (D) cells, and no significant difference was observed between TEAD4‐siRNA cells with control cells. Statistical analyses were presented in the right panel. All experiments were performed 3 times

Our clinical results and analyses revealed a possible tumour‐promoting role of TEAD4 in LAD, and we next aimed to confirm its molecular effect in the corresponding tumour cells. Firstly, we investigated the mRNA and protein levels of TEAD in 6 human LAD cell lines (A549, SPC‐A1, NCI‐H1299, H1650, PC9 and H1975), one LSCC cell line (NCI‐H1703) and one normal bronchial epithelial cell line (HBE) as control. The qPCR (Figure 3A) and Western blot (Figure 3B) results indicated distinct expression patterns of TEAD4 in those cell lines. Specifically, all NSCLC cell lines showed higher TEAD4 levels than that of HBE cells. H1299 and SPC‐A1 cell lines, the two with highest TEAD4 protein level, were then subjected to gene‐silencing experiments using specific siRNA (Figure 3C,D). The proliferation capacity was evaluated by MTT assay and colony formation assay; both showed no significant alteration upon TEAD4‐siRNA transfection (Figure 3E,F). The Edu staining experiments further verified the little effect of TEAD4 on LAD cell proliferation (Figure 3G). Additionally, we tested cell cycle in TEAD4 knockdown cells by flow cytometry, and neither H1299 nor SPC‐A1 showed significant change in cell cycle pattern (Figure 4A,B). As no evidence on the effect of TEAD4 in cell proliferation or growth, we then analysed cell apoptosis process. As expected, no apoptotic effect of silencing TEAD4 was observed in the two LAD cell lines (Figure 4C,D).

### 3.4 TEAD4 knockdown suppresses lung adenocarcinoma metastasis

**Figure 5:**
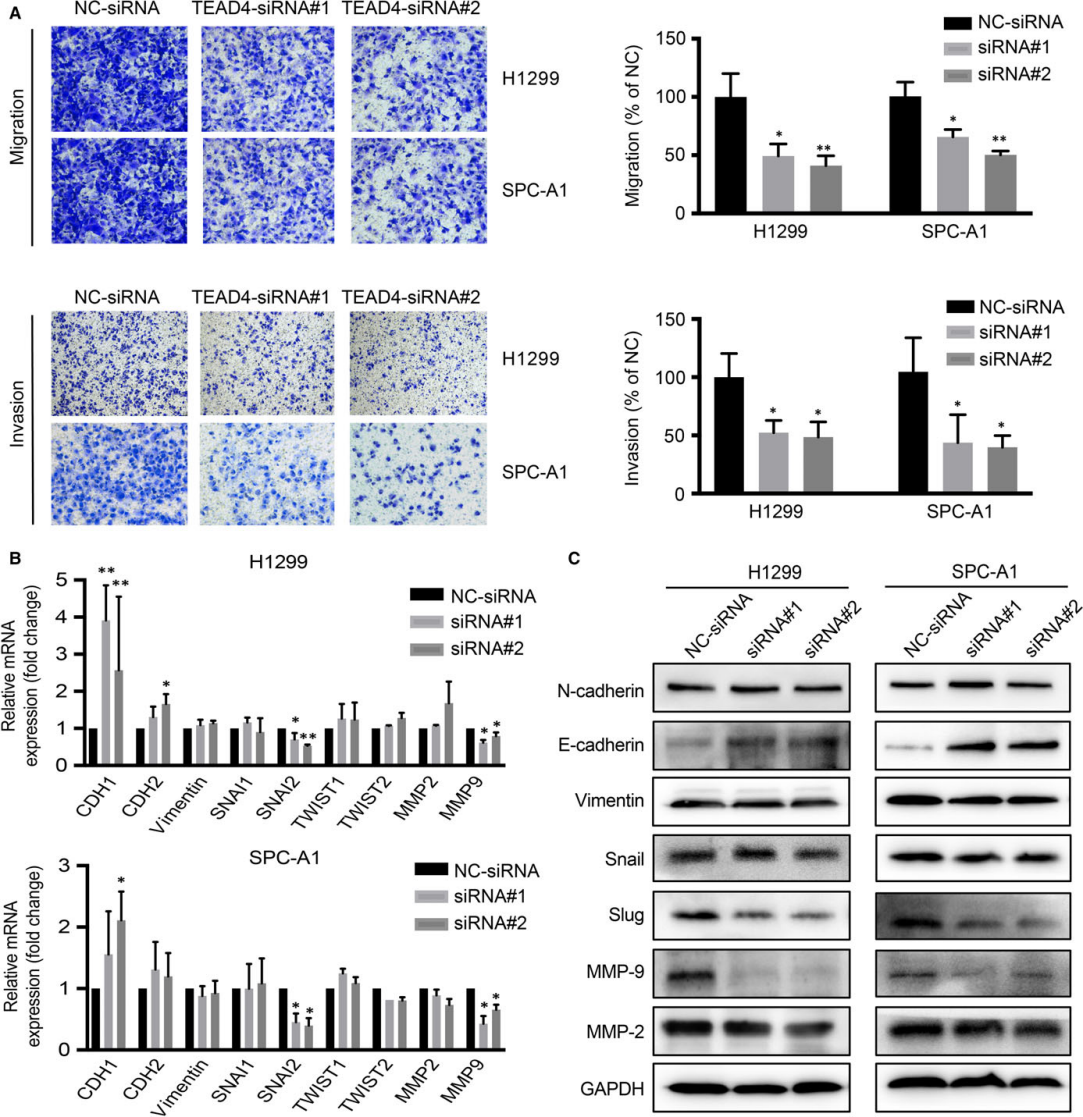
Knocking down of TEAD4 attenuates tumour metastatic capacities of LAD Cell migration and invasion of transfected cells were tested by transwell assay; TEAD4 knockdown cells showed a decreased migration and invasion capacity in both H1299 and SPC‐A1 cells. B, Epithelial‐mesenchymal transition (EMT) markers (CDH1, CDH2, Vimentin, SNAI1, SNAI2, TWIST1 and TWIST2) and MMPs (MMP2 and MMP9) associated with metastasis were detected by qRT‐PCR. C, EMT molecules (N‐cadherin, E‐cadherin, Vimentin, Snail and Slug) and MMPs (MMP2 and MMP9) were examined by Western blot with corresponding antibodies. Data obtained from at least 3 independent experiments. *P <* *.05 and **P <* *.01 compared to negative control (NC) group

We next explored the regulatory role of TEAD4 in LAD cellular motility via transwell assays, which revealed a positive effect of TEAD4 on promoting the migration process of H1299 and SPC‐A1 cells (Figure 5A). Besides, the invasion capacity of H1299 and SPC‐A1 cells was assessed by Matrigel‐transwell strategy. As expected, silencing TEAD4 presented a ~50% invasion decrease in the two LAD cells lines (Figure 5B). Furthermore, we evaluated the expression changes of EMT markers by qPCR (Figure 5C) and Western blot (Figure 5D). The results demonstrated a significant increase in E‐cadherin expression in siRNA‐transfected H1299 and SPC‐A1 cells. In contrast, a lower level of Slug was detected in TEDA4‐silencing cells compared with that in control cells. These data suggested that TEAD4 promoted EMT process in LAD cells. Additionally, the expression patterns of certain matrix metalloproteinases (MMPs) in transfected LAD cells were monitored, revealing a possible pro‐metastatic role of MMP9 downstream of TEAD4 in LAD progression.

### 3.5 Regulation of TEAD4 by miR‐6839‐3p

**Figure 6:**
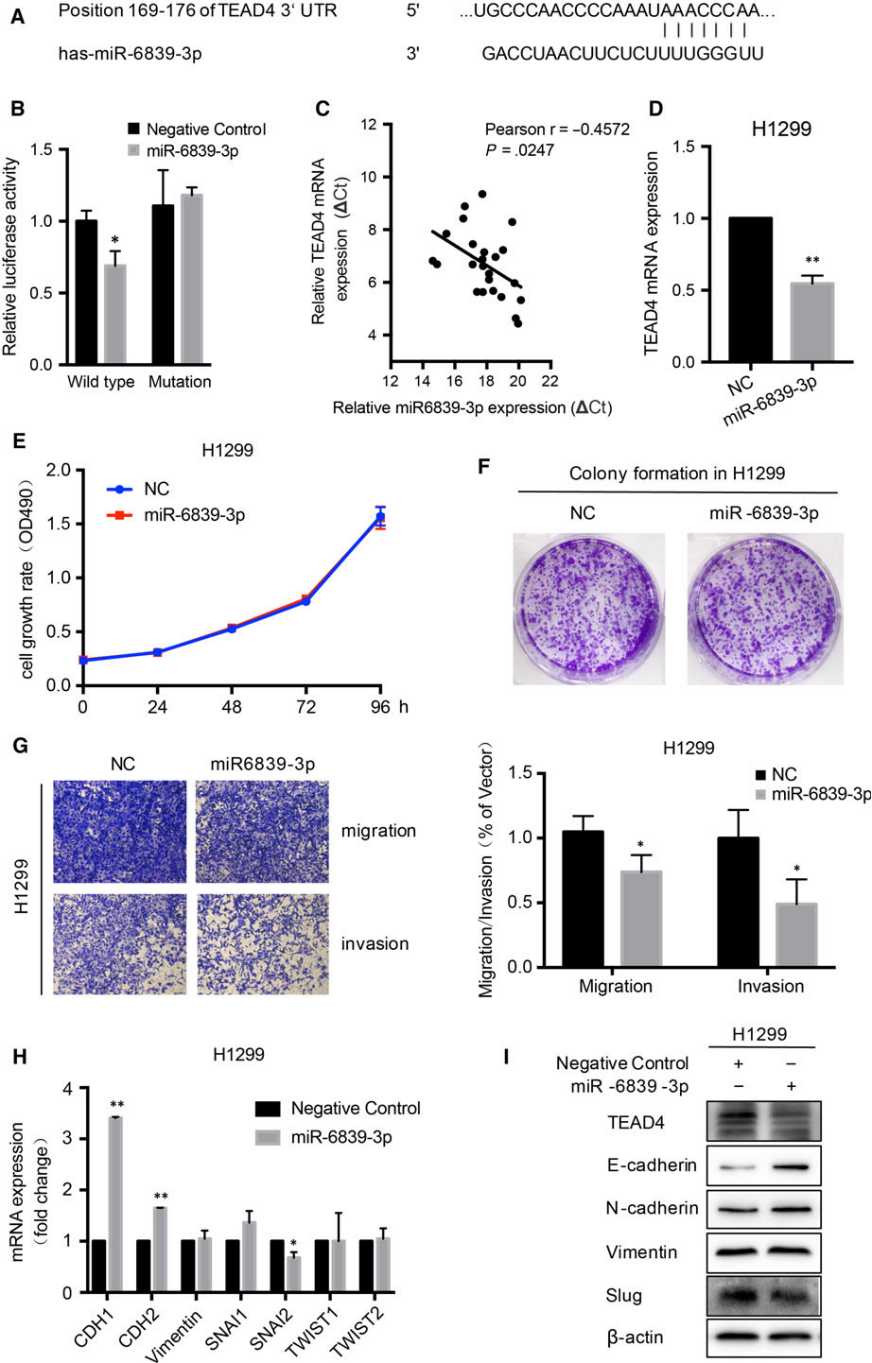
MiR‐6839‐3p is an antimetastatic miRNA in LAD by directly targeting TEAD4. A, The putative miR‐6839‐3p binding site was in the 3′UTR of TEAD4 as predicted by TargetScan (http://www.targetscan.org). B, The luciferase activity of constructs containing the wild‐type binding site was inhibited by miR‐6839‐3p, but miR‐6839‐3p did not affect the luciferase activation in the construct encompassing mutated binding site in TEDA4 3′UTR (WT, wide‐type of miR‐6839‐3p binding sequence in TEDA4 3′UTR; mutation, the binding site was mutated). C, A negative correlation between expression of miR‐6839‐3p and TEAD4 was detected in 24 pairs of LAD tissues (*r *= −.4572, *P *=* *.0247). D, TEAD4 mRNA expression was down‐regulated by overexpressing miR‐6839‐3p in H1299 cells. MTT (E) and colony formation (F) assays showed no significant effect of miR‐6839‐3p on cell proliferation. G, Cell migration and invasion were impaired in LAD cells after overexpressing miR‐6839‐3p. Quantitative RT‐PCR (H) and Western blot (I) were used to detect the mRNA and protein levels of EMT‐related molecules. All experiments were performed at least 3 times. *P <* *.05 and **P <* *.01 compared to negative control (NC) group

Finally, we aimed to map upstream miRNA regulators of TEAD4. Several potential miRNAs including miR‐6839‐3p, miR‐6742‐3p, miR‐1343‐3p, miR‐6855‐5p, miR‐4269 and miR‐375 might target TEAD4 according to TargetScan (http://www.targetscan.org). However, qRT‐PCR screening showed only miR‐6839‐3p had a significant regulatory role on the expression of TEAD4 in H1299 cells (Figure S1). The targeting sites on 3′‐UTR of TEAD4 by miR‐6839‐3p were therefore listed (Figure 6A). To test whether TEAD4 is a directed target of miR‐6839‐3p, we performed the luciferase assay. As results, the relative luciferase activity of construct encompassing the wild‐type binding site in TEAD4 3′‐UTR was highly inhibited by miR‐6839‐3p. But miR‐6839‐3p had no effect on the mutated sequence of the binding site (Figure 6B). To validate our findings, a total of 24 LAD tissues were enrolled to investigate the association between miR‐6839‐3p and TEAD4, which showed a negative correlation between their expression levels (Figure 6C, *r *= −.4572, *P *=* *.0247). Additionally, both the mRNA and protein levels of TEAD4 were significantly decreased after overexpressing the miR‐6839‐3p mimic in cultured LAD cells (Figure 6D). Of note, although miR‐6839‐3p showed little effect on LAD cell proliferation (Figure 6E,F), its overexpression remarkably impaired cell migration and invasion capacities according to the transwell results (Figure 6G). Consistently, the mRNA and protein expressions of EMT markers such as E‐cadherin and Slug were consequently changed upon miR‐6839‐3p transfection in LAD cells (Figure 6H,I). Therefore, we revealed a novel signalling axis of miR‐6839‐3p‐TEAD4‐E‐cadherin/Slug and its role in the progression of LAD.

## 4 DISCUSSION

Accumulating evidence showed that mutations and altered expressions of components in Hippo pathway were involved in tumour development.^22^ As a core protein of the emerging Hippo signalling pathway in mammals, YAP promotes cellular growth, inhibits cell apoptosis and accelerates EMT.^23^, ^24^ Of note, YAP's tumorigenesis activity by transcriptional regulation depends largely on its physical interaction with transcriptional factors TEADs.^25^ The expression of all four TEAD members was detected in lung adenocarcinoma tissues compared with adjacent normal ones in this study, indicating the up‐regulated expression of TEAD3 and TEAD4 in tumour (Figure S1). However, the function of TEAD3 is mainly focused on the embryos^26^ while TEAD4 might play a key role in tumorigenesis.

TEAD4 has been reported to modulate tumour progression through various pathways in several tumour types. For example, it can induce expression of connective tissue growth factor (CTGF) and c‐Myc proteins and therefore promote tumour proliferation of colorectal adenocarcinoma^27^ and gastric adenocarcinoma.^8^ Similarly, in oral squamous cell carcinoma, TEAD4 ensures the high levels of cyclins and cyclin‐dependent kinases (CDKs), and subsequently accelerate cell cycle.^28^ However, in the current study, we did not find any significant effect of TEAD4 on cell proliferation of lung adenocarcinoma (LAD) cells. Neither the cell cycles nor cell apoptosis were statistically altered by silencing TEAD4. It seems that TEAD4 functions distinctively between digestive system tumours and lung cancers. This difference can be partially explained by the fact that each cell type possesses specific transcription profiles, and the transcriptional regulation is therefore diverse among tumours. Further genomic or proteomic studies in TEAD4‐silencing or TEAD4‐overexpressing lung cancer cells may better illuminate our hypothesis.

Although do not affect cell proliferation or apoptosis, TEAD4 indeed shows an up‐regulated expression in clinical LAD tissues from both mRNA and protein levels. Besides, LAD patients with higher TEAD4 expressions are characterized with higher recurrence risk and poorer overall survival. Therefore, we were interested in exploring whether and how TEAD4 promotes LAD progression. According to our data, TEAD4 knockdown dramatically attenuated cell migration and invasion capacities, which was consistent with clinical findings. Considering its eligible effect on tumour proliferation and apoptosis, the pro‐metastatic role of TEAD4 seems more significant as revealed by transwell assay. Moreover, we found that several mesenchymal phenotype biomarkers including Vimentin, Slug and MMP9 were positively regulated by TEAD4. Interestingly, although predominantly functioning downstream of YAP activation, a recent study described a YAP‐independent effect of TEAD4 in promoting Vimentin expression,^29^ consequently inducing the EMT process. Accordingly, the EMT alterations in LAD cells may be caused by both YAP‐dependent and YAP‐independent signalling pathways.

Besides downstream effectors, upstream regulation of oncogenes is attracting more and more attention. A genomic study reported that TEAD4 was significantly hypomethylated in gastric cancer (GC) tissues.^5^ They provided insights on the correlations between epigenetic modifications of TEAD4 with GC clinical outcome. Another important upstream regulator prior to mRNA translation is the microRNAs (miRNAs), which are small non‐protein‐coding RNAs that function as negative gene regulators. The clinical significance of miRNAs has been well‐acknowledged in tumour diagnosis, staging, prognosis and therapy response.^30^ A previous study demonstrated that TEAD4 was negatively regulated by miR‐1343‐3p and miR‐4269 in gastric cancer.^8^ However, after transfecting miR‐1343‐3p or miR‐4269 in human LAD cell lines, there was no significant regulation on TEAD4 expression according to our data (Figure S2). This difference may due to the tissue and cell specificity of miRNA, and thus, we sought to uncover novel TEAD4‐targeted miRNA in LAD. Firstly, we identified that position 169‐176 in the 3′UTR of TEAD4 can bind with miR‐6839‐3p based on the nucleotide sequence. Then, we proved miR‐6839‐3p targeted TEAD4 according to the luciferase activity assays. Secondly, the RNA level of TEAD4 was negatively correlated with that of miR‐6839‐3p in LAD tissues. Consistently, miR‐6839‐3p showed a significant lower level in LAD tissues than in normal lung tissues, indicating it may serve as a tumour suppressor. Thirdly, miR‐6839‐3p transfection in LAD cells led to a decreased TEAD4 level and subsequently inhibited the expression of EMT molecules. Finally, miR‐6839‐3p transfection restrained tumour metastasis without affecting proliferation of LAD cells, which was quite similar to the phenotypes of TEAD4 knockdown cells. Taken together, our data initially demonstrated the role of miR‐6839‐3p in suppressing LAD progression by targeting TEAD4.

## 5 CONCLUSIONS

TEAD4 is up‐regulated in lung adenocarcinomas and correlated with unfavourable clinical outcome. A high TEAD4 level results in excessive transcription and expression of EMT proteins and therefore promotes tumour metastasis. miR‐6839‐3p is a novel tumour‐suppressor functions by targeting TEAD4 in lung adenocarcinoma.

## CONFLICT OF INTEREST

The authors confirm that there are no conflict of interests.
