# Corrigendum

**DOI:** 10.1111/jcmm.15304

**Published:** 2020-07-07

In Zhang et al,^1^ the published article contains errors in Figure 3F and Figure 5A. The correct figures are shown below. The authors confirm all results and conclusions of this article remain unchanged.

**FIGURE 3:**
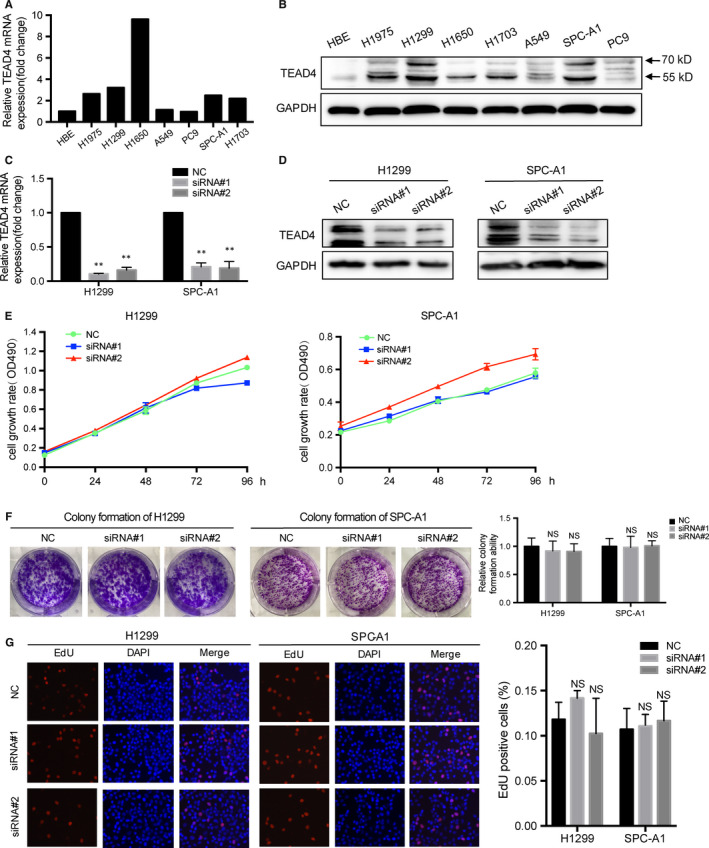
Silencing TEAD4 exerts no effect on the proliferation of LAD cells. Quantitative PCR A, and Western blot (B) were used to measure the TEAD4 levels in human bronchial epithelial cell line (HBE) and NSCLC cell lines (A549, H1975, H1299, H1650, A549, PC9, SPC‐A1 and H1703), indicating higher TEAD4 levels in NSCLC cells. The H1299 and SPC‐A1 cells were transfected with TEAD4‐siRNA, and transfection efficiencies were verified by qRT‐PCR (C) and Western blot (D), respectively. Silencing TEAD4 showed no significant effect on cellular proliferation as revealed by MTT (E), colony formation (F) and Edu staining (G) assays. The data were represented as the mean ± SD acquired from three repeated experiments

**FIGURE 5:**
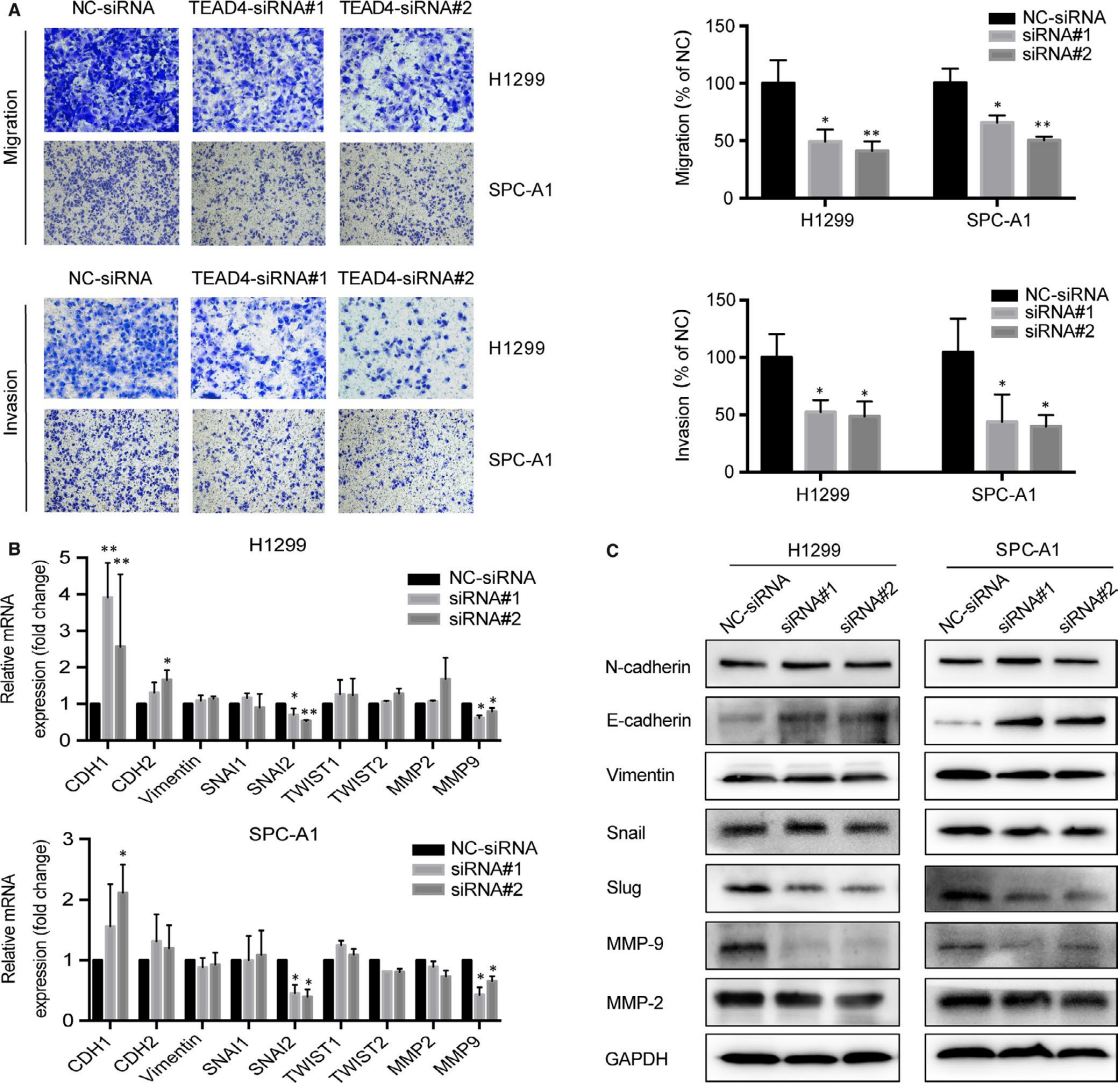
Knocking down of TEAD4 attenuates tumour metastatic capacities of LAD cells. A, Cell migration of transfected cells was tested by transwell assay; TEAD4‐knockdown cells showed a decreased migration capacity in both H1299 and SPC‐A1 cells. B, The cell invasion was measured by Matrigel transwell assay, revealing the possible role of TEAD4 in promoting LAD invasion. C, Epithelial‐mesenchymal transition (EMT) markers (CDH1, CDH2, Vimentin, SNAI1, SNAI2, TWIST1 and TWIST2) and MMPs (MMP2 and MMP9) associated with metastasis were detected by qRT‐PCR. D, EMT molecules (N‐cadherin, E‐cadherin, Vimentin, Snail and Slug) and MMPs (MMP2 and MMP9) were examined by Western blot with corresponding antibodies. Data obtained from at least three independent experiments. *P < .05 and **P < .01 compared with negative control (NC) group

The authors apologize for the inconvenience this may cause.
