# Supplementary material for: TEAD4 exerts pro‐metastatic effects and is negatively regulated by miR6839‐3p in lung adenocarcinoma progression
Source: J Cell Mol Med. 2018 Apr 18;22(7):3560–71. doi: 10.1111/jcmm.13634 (PMC6010880; doi:10.1111/jcmm.13634)
Supplement: Supplementary file 1 [file JCMM-22-3560-s001.docx]

**Supplemental File**

Title: MiR6839-3p restrains epithelial–mesenchymal transition and metastasis of lung adenocarcinoma by suppressing transcriptional enhancer associate domain protein 4

Contents:

Table S1.

Table S2.

Figure S1.

Figure S2.

Figure S3.

Table S1. The primers used for qPCR analysis.

| Primer Name | Primer Sequence 5’-3’ |
| --- | --- |
| TEAD4-F | TCCACGAAGGTCTGCTCTTT |
| TEAD4-R | GTGCTTGAGCTTGTGGATGA |
| Actin-F | TGACGTGGACATCCGCAAAG |
| Actin-R | CTGGAAGGTGGACAGCGAGG |
| SNAI1-F | ACTGCAACAAGGAATACCTCAG |
| SNAI1-R | GCACTGGTACTTCTTGACATCTG |
| SNAI2-F | TGTGACAAGGAATATGTGAGCC |
| SNAI2-R | TGAGCCCTCAGATTTGACCTG |
| TWIST1-F | GTCCGCAGTCTTACGAGGAG |
| TWIST1-R | TGAATCTTGCTCAGCTTGTCC |
| TWIST2-F | CAAGCTGAGCAAGATCCAGA |
| TWIST2-R | ATTGTCCATCTCGTCGCTCT |
| CDH1-F | ATTTTTCCCTCGACACCCGAT |
| CDH1-R | TCCCAGGCGTAGACCAAGA |
| CDH2-F | TGCGGTACAGTGTAACTGGG |
| CDH2-R | GAAACCGGGCTATCTGCTCG |
| VIM-F | AGTCCACTGAGTACCGGAGAC |
| VIM-R | CATTTCACGCATCTGGCGTTC |
| MMP2-F | TACAGGATCATTGGCTACACACC |
| MMP2-R | GGTCACATCGCTCCAGACT |
| MMP9-F | AGACCTGGGCAGATTCCAAAC |
| MMP9-R | CGGCAAGTCTTCCGAGTAGT |
| miR6839-3p | CGCGTTGGGTTTTCTCTTCAATCCAG |
| U6 | CGAATTTGCGTGTCATCCT |

Table S2. The information about the antibodies in our study.

| Antibody | Vender | Cat.No. | Dilution | Host Species | Application |
| --- | --- | --- | --- | --- | --- |
| TEAD4 | Sigma-Aldrich | 7004 | 1:1000 | Mouse | IHC,WB |
| β-actin | CST | 4970 | 1:1000 | Rabbit | WB |
| N-cadherin | CST | 3195 | 1:1000 | Rabbit | WB |
| E-cadherin | CST | 13116 | 1:1000 | Rabbit | WB |
| Vimentin | CST | 5741 | 1:1000 | Rabbit | WB |
| Snail | CST | 3879 | 1:1000 | Rabbit | WB |
| Slug | CST | 9585 | 1:1000 | Rabbit | WB |
| MMP-2 | CST | 40994 | 1:1000 | Rabbit | WB |
| MMP-9 | CST | 13667 | 1:1000 | Rabbit | WB |

CST: Cell Signaling Technology; WB: Western blotting; IHC: Immunohistochemical staining.

Figure S1.


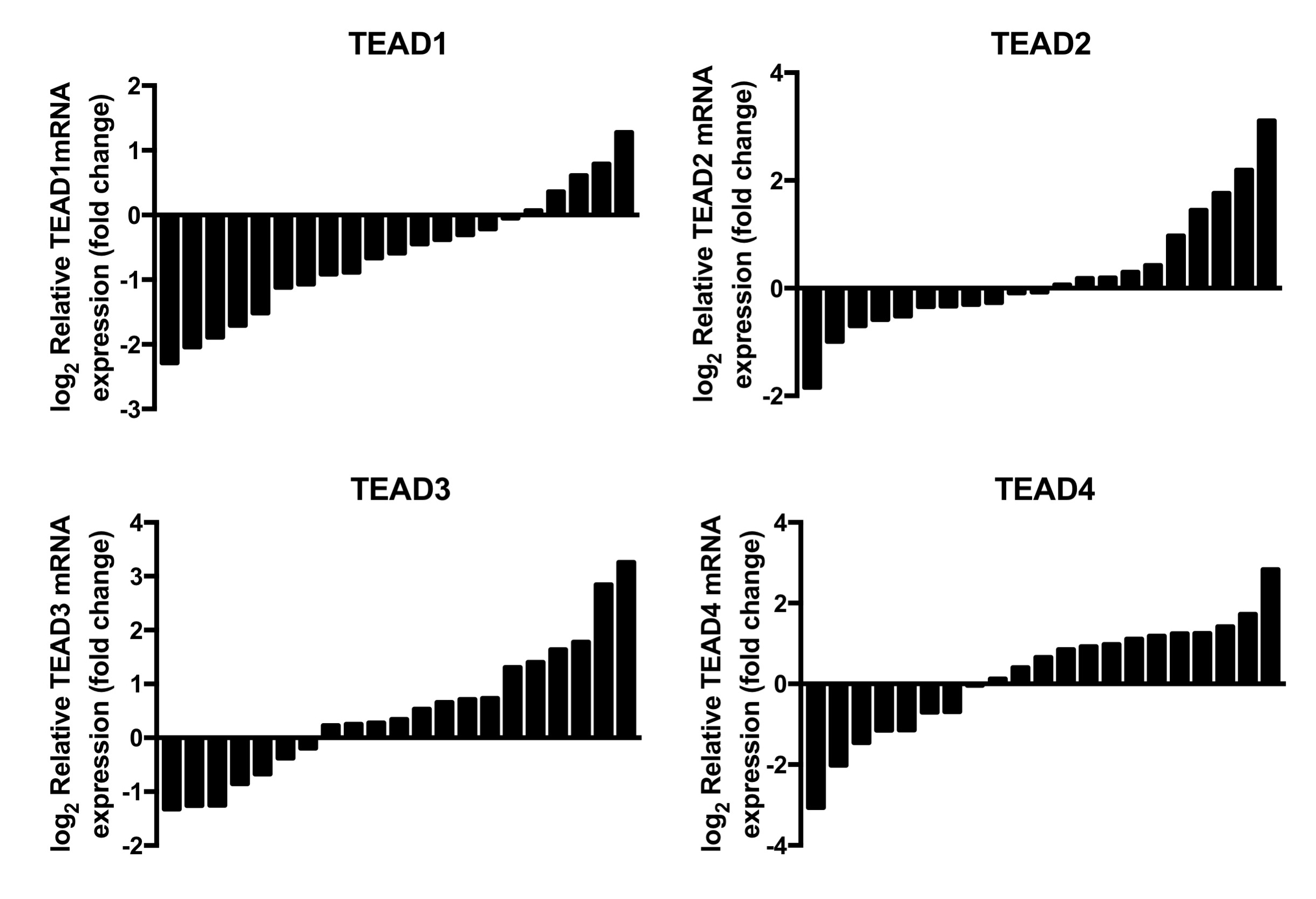


**Figure S1.** The expression of TEAD1-4 in lung adenocarcinoma tissues.

Real-time PCR quantification of TEAD1-4 mRNA levels in lung adenocarcinoma (LAD) tissues and matched adjacent nontumoral tissues (n=21)

Figure S2.

**Figure S2.** Effects of predicted miRNAs on the expression of TEAD4.

The LAD H1299 cells were transfected with different miRNA mimics, and their impact on TEAD4 RNA levels were tested by PCR. None of those showed statistical significance.

Figure S3.

**Figure S3.** miR6839-3p mimics transfection efficiency.

The H1299 cells were transfected with miRNA mimics or negative control (NC), and the expression level of miR6839-3p was detected by PCR. ***p*<0.01 compared to negative control (NC) group.
